# Effectiveness of public health spending: Investigating the moderating role of governance using partial least squares structural equation modelling (PLS-SEM)

**DOI:** 10.1186/s12961-024-01159-x

**Published:** 2024-07-08

**Authors:** Wa Ntita Serge Kabongo, Josue Mbonigaba

**Affiliations:** School of Accounting, Economics and Finance, University of KwaZulu-Natal, 15 University Road, Westville, 4001 Durban South Africa

**Keywords:** Public health spending, Population health outcomes, Moderation analysis, Governance, PLS-SEM

## Abstract

**Background:**

The link between public health spending (PHS) and population health outcomes (PHO) has been extensively studied. However, in sub-Saharan Africa (SSA), the moderating effects of governance in this relationship are little known. Furthermore, studies have focused on mortality as the main health outcome. This study contributes to this literature by investigating the moderating role of governance in the relationship by simultaneously assessing three dimensions of governance (corruption control, government effectiveness and voice accountability) using disability-adjusted life years (DALYs) as a measure of outcomes.

**Methods:**

The study applies the two-stage moderation approach using partial least squares structural equation modelling (PLS-SEM) to panel data from 43 SSA nations from 2013 to 2019. The study also uses domestic general government health expenditure (DGGHE) as an independent variable and disability-adjusted life years (DALY) as the dependent variable in this relationship.

**Results:**

The analysis reveals that DGGHE affects DALY negatively and that governance improves the effect of DGGHE on DALY, with bigger improvements among countries with worse governance.

**Conclusion:**

These findings provide evidence that good governance is crucial to the effectiveness of PHS in SSA nations. Sub-Saharan Africa (SSA) countries should improve governance to improve population health.

## Background

Improving population health has been extensively emphasized in health-related literature [5, 7, 24, 65]. However, SSA nations continue to have poor population health and lag behind in reaching universal health coverage and health-related sustainable development targets [71]. In their efforts to improve the health of their citizens, SSA nations frequently rely on public health spending (PHS) to reduce catastrophic healthcare costs and achieve universal health coverage [59, 72]. PHS is one of the primary sources of funding in SSA nations for reaching Sustainable Development Goal 3 (SDG 3), which seeks to boost the health of a country’s overall population [42]. SSA nations no longer receive substantial external health aid [70], implying a higher share of out-of-pocket health expenditure. PHS may be a tool for curbing the negative effects of the high proportion of out-of-pocket health expenditures in these nations. Accessing health services via out-of-pocket payment restricts access and pushes 1.5% of the population below the poverty line [71].

SSA and developing nations generally face contradictory findings regarding leveraging PHS to improve their populations’ health [40]. This situation stems from the fact that research does not similarly capture essential factors affecting the effectiveness of PHS. The literature indicates that healthcare, the channel through which PHS impacts population health outcomes (PHO), accounts for only about 28% of the PHO improvement [55]. In SSA, governance factors, among other factors affecting health outcomes, are particularly important. Two perspectives explain the negative effects of poor governance on the health sectors of SSA nations. First, the lack of proper governance may lead to the ineffectiveness of PHS in improving health status via fiscal misallocations and, second, the negative externalities of poor governance via unfair access [61]. For example, bribes paid may facilitate an unfair health services access in favour of the least-to-benefit people, with consequences of lower health output, as some studies pointed out [44].

From the above, a question arises about the impact that poor governance may have on the effectiveness of PHS in the SSA region [67], where health funding remains a severe constraint [14, 48]. In 2017, the region’s per capita health spending was US$84 , compared with the global average of US$1065, and the government spending on health as a share of general government spending swayed around 7.2% despite the 2011 Abuja commitment by African countries to spend 15% of the state budget on health [69].

Good governance is expected to enhance the effectiveness of PHS on health outcomes in SSA nations, although limited evidence exists to this effect. Thus, this study seeks to fill this gap by examining how the country’s governance moderates the relationship between the PHS and PHO, specifically between domestic general government health expenditure (DGGHE) and disability-adjusted life years (DALY). On the one hand, DGGHE reflect outlays earmarked for health maintenance, restoration or enhancement by the government from domestic sources [70]. On the other hand, DALY reflects a more inclusive population measurement based on mortality and morbidity rates [21]. The study also contributes knowledge by using three governance dimensions (corruption control, government effectiveness and voice accountability), evaluated simultaneously as moderators, and their possible interactions to affect the link between PHS and PHO. To the best of our knowledge, this is the first study in SSA to simultaneously evaluate three governance components as moderators of the relationship between DGGHE and DALY. The study addresses two questions: (1) Is DGGHE related to DALY? (2) Does good governance increase the impact of DGGHE on DALY? This study aims to evaluate whether and to what extent higher DGGHE improves DALY in SSA countries and whether the quality of governance in these countries changes this relationship. To answer the questions above, the partial least squares structural equation modelling (PLS-SEM) approach is applied to panel data from 43 SSA nations from 2013 to 2019 using publicly accessible data.

The remaining sections of this study are organized as follows: The literature review and creation of hypotheses are covered briefly in the “Literature review and hypothesis development” section. The methodology and data are discussed in the “Methods” section. The “Results” section contains the findings of the study. The discussion is carried on in the “Discussion”s. Conclusions and suggestions for further research are included in the final section.

## Literature review and hypothesis development

### Literature on PHS and PHO

Since the 1990s, macro health expenditures have gained popularity. The relationship between health expenditures and population health has been one of the most studied areas of health [18, 41, 60]. Limited research has evaluated the PHS–PHO relationship in developing regions, notably SSA, where results have been inconclusive. Some SSA studies have shown that PHS improves PHO [23, 51, 52, 61], but others have not [2, 18, 33, 57]. Most of these studies have solely examined the ultimate impact of the PHS on PHO as evaluated by mortality-based indicators, such as under-5-years mortality and life expectancy. More investigation is needed to unveil the nature of the link between PHS and PHO in SSA when a more comprehensive measure of DALY is used and new investigation perspectives explored. As indicated earlier, certain factors, such as the country’s governance, can influence this relationship, which may alter the direction and size of the predictors’ influence.

Studies on governance have been carried out from three theoretical perspectives: the conceptualization of the quality and quantity of government, the scope and type of quality of the government and the direct or indirect role of government quality in the causal mechanism [39]. This study focuses on the third perspective, where the moderating effect of governance on the effectiveness of PHS is investigated.

Literature on the moderating effect of governance on the effectiveness of PHS is lacking in SSA. Studies that have explored the subject include, for example, Rajkumar and Swaroop [61]. They found that governance quality affects the impact of PHS on policy outcomes such as child education and health. The study results suggested that, in poorly governed countries, PHS had little impact on the outcomes, whereas in countries with good governance, PHS on healthcare significantly affected child education and child mortality rates.

Similarly, investigating the effect of government health spending on PHO measured by infant and under-5-years mortality, the study by Farag et al. [16] found differing findings in the presence and absence of government effectiveness. The study concluded that improvements in government effectiveness enhanced the effect of government health spending [16]. Another study by Kim and Wang [39] investigated the moderating effect of the quality of government on the relationship between the quantity of governance measured by PHS and PHO measured by mortality-based indicators (infant, under-5-year, and maternal mortality and life expectancy). The study also found differing results in the presence and absence of the quality of governance, notably that the quality of governance played a role in moderating the relationships between the quantity of governance and the PHO [39].

### Hypothesis development

The health literature reveals that PHS is one tool to improve PHO, reduce catastrophic healthcare spending and advance universal health coverage [59, 72]. Various studies have empirically demonstrated that PHS improves PHO. For example, Anyanwu and Erhijakpor [4] examined the effectiveness of total health expenditure (THE) and public health expenditures (PHE) on PHO as proxied by under-5-year and infant mortality rates across African countries using panel data and two-stage ordinary least squares estimation. Their findings indicated that both THE and PHE per capita had a significant negative effect on under-5-year and infant mortality rates, with THE elasticities of −0.21 and −0.22 for under-5-year and infant mortality, respectively, and PHE elasticities of −0.25 and −0.21 for under-5-year and infant mortality, respectively [4]. Grekou and Perez [22] conducted a study covering 2000–2011 to determine the factors contributing to child mortality in SSA [22]. They used panel data with fixed and random effects and instrumental variables and found a positive relationship between PHS and child and under-5-year mortality. Novignon and Lawanson [51] used fixed and random effects panel data regression models and data from 45 SSA countries from 1995 to 2011 to examine the relationship between healthcare spending and child health outcomes (infant, under-5-year and neonatal mortality). Their findings suggested a negative and significant relationship with elasticities of −0.11, −0.15 and −0.08 for infant mortality, under-5-year mortality and neonatal mortality, respectively [51]. The discussion above stresses the importance of PHS in influencing PHO. From the discussion, it follows that the following hypothesis on the link between PHS (measured by DGGHE) and PHO (measured by DALY) can be formulated:

- H1: DGGHE has a negative effect on DALY.

In an era of tighter public sector resources, policy-makers must demonstrate strong results from public social spending, which usually requires good governance. Good governance enhances economic performance and supports income growth [29]. Some studies in the development literature have highlighted the impact of governance quality on social outcomes, including population health [18, 58]. For example, a cross-country study by Gupta et al. [23] suggested that public spending on healthcare only has an insignificant effect on child mortality rates. In contrast, corruption itself had a significant negative impact. One idea shared by studies investigating the effects of governance on PHS is that, in countries where governance is poor, public resources suffer from leakages and fail to translate into social investments that could result in desirable social outcomes such as better child health. Kaufmann et al. [36] and Kaufman et al. [34] showed that governance indicators such as voice accountability, political stability, government effectiveness and corruption, among others, strongly negatively impacted infant mortality. Lin et al. [43] applied a semi-parametric generalized additive mixed model to study governance’s influence on child mortality in 149 countries from 1996 to 2020. They utilized six dimensions of governance (political stability, control of corruption, government effectiveness, voice accountability, regulatory quality and the rule of law). Their findings reveal that improved governance reduces child mortality via its distribution of health-related programmes and response to the citizens’ needs. Emamgholipour and Asemane [15] found similar results. They studied the effect of governance indicators on under-5-year mortality in 27 Organization for Economic Cooperation and Development (OECD) countries from 1996 to 2012, employing the generalized method of moments approach. The result revealed that increased governance quality reduces under-5-year mortality in OECD [15]. Therefore, drawing on these arguments, the study proposes the following hypothesis:

- H2: The country’s governance significantly influences the relationship between DGGHE and DALY.

## Methods

### Data sources, constructs and indicators

To test the proposed hypotheses, this paper used a panel of 43 SSA countries [Angola, Benin, Botswana, Burkina Faso, Burundi, Cabo Verde, Cameroon, Central African Republic, Chad, Comoros, Cote d’Ivoire, the Democratic Republic of Congo (DRC), the Republic of Congo (Congo, Rep.), Equatorial Guinea, Eritrea, Eswatini, Ethiopia, Gabon, Gambia, The, Ghana, Guinea, Guinea-Bissau, Kenya, Lesotho, Liberia, Madagascar, Malawi, Mali, Mauritania, Mozambique, Namibia, Niger, Nigeria, Rwanda, Sao Tome and Principe, Senegal, Sierra Leone, South Africa, Tanzania, Togo, Uganda, Zambia and Zimbabwe] from 2013 to 2019 subject to data availability. Data were drawn from the Institute of Health Metrics and Evaluation (IHME), Global Health Expenditure (GHE), World Governance Index (WGI) and World Development Index (WDI) databases. The data on disability-adjusted life years (DALY) were extracted from the IHME. The DGGHE data were collected from the GHE. The study used data on the first three of the following six dimensions of governance indicators: control of corruption, government effectiveness, voice accountability, political stability and absence of violence/terrorism, the rule of law and regulatory quality [35]. Each country is assigned a score (value) ranging from −2.5 to +2.5 for each of the four indicators in this database. A score of −2.5 indicates a very low rating, while a score of +2.5 indicates a very high rating [37]. The use of governance indicators arouses considerable debate among researchers about which indicators to include, as each indicator embeds different governance information [8]. This study selected the first three governance dimensions on the basis of the data availability, relevance to the SSA context and the multicollinearity consideration.

A significant variable that should have been incorporated into this analysis is violence, considering its well-documented effects on health outcomes and government expenditure on healthcare in SSA. The region has experienced some forms of violence rooted in political, ethnic, religious, economic and governance conflicts. Nevertheless, out of the nations studied, only five – DRC, Kenya, Nigeria, Burundi and Zimbabwe [32, 68] and [45] – experienced noteworthy levels of violence, although limited in nature in some cases. In most cases, non-open-wars types of violence, such as gender-based violence (GBV) and protest-related violence [9, 10], are not explicitly and officially reported [38, 56]. Although it was a relevant variable, it was not available for inclusion in the analysis in most countries in the study. However, all accessible data were publicly available and did not require ethical clearance. The University of KwaZulu-Natal Ethics Committee issued the study’s ethics exemption letter.

Using their yearly indicators, the study built first-order governance constructs for those three components. Then, a second-order governance construct was built using the constructs of the three first-order components. Other study variables were first-order constructs built on the basis of their yearly indicators in the study dataset. The control variables – female education (FEMED), the incidence of malaria (MALA), the incidence of tuberculosis (TB) and the prevalence of human immunodeficiency virus (HIV) – were collected from WDI and selected on the basis of previous research and on the availability of the data. In Table 1, each construct’s composition is described.

**Table 1:** Latent variables and their manifesting variables

Constructs	Indicators	Description
DALY	DALY, total	Disability-adjusted life years, all causes, both (per 100,000 people)
DGGHE	DGGHE, total	DGGHE per capita, PPP (cost in 2017 international $)
Female education	Female education	School enrollment, primary, female (% gross)
	Control of corruption	Control of corruption: estimate
Governance	Government effectiveness	Government effectiveness: estimate
	Voice accountability	Voice and accountability: estimate
Prevalence of HIV/AIDS	Prevalence of HIV/AIDS	Incidence of malaria (per 1000 population at risk)
Incidence of malaria	Incidence of malaria	Incidence of HIV, all (per 1000 uninfected population)
Incidence of tuberculosis	Incidence of tuberculosis	Incidence of tuberculosis (per 100,000 people)

AIDS, acquired immune deficiency syndrome

Sources: WDI, WGI, IHME and GHE databases

### Estimation method

To validate the study constructs and test the hypotheses, this study applied PLS-SEM rather than covariance-based structural equation modelling (CB-SEM) for the following reasons: PLS-SEM has low constraints on measurement scales, sample size and residual distributions; PLS-SEM analysis does not presume complete independence of variables, resulting in more accurate results; and PLS-SEM handles missing data and is robust against data skewness and the omission of an independent variable [26, 27]. Interaction terms are used to conduct moderator analyses in PLS-SEM.

Three main approaches are used to generate the interaction terms: product-indicator, orthogonalizing and two-stage approaches [30]. This study used the two-stage approach proposed by Henseler and Fassott [31] because it overcomes the limitations of the product-indicator and orthogonalizing approaches. The literature on moderation provides a detailed discussion of these approaches [25, 28, 30, 31]. This study selected PLS-SEM for the above reason and because it has been implemented in numerous economic and public health research studies [3, 6, 19, 50, 54, 62].

**Fig. 1:**
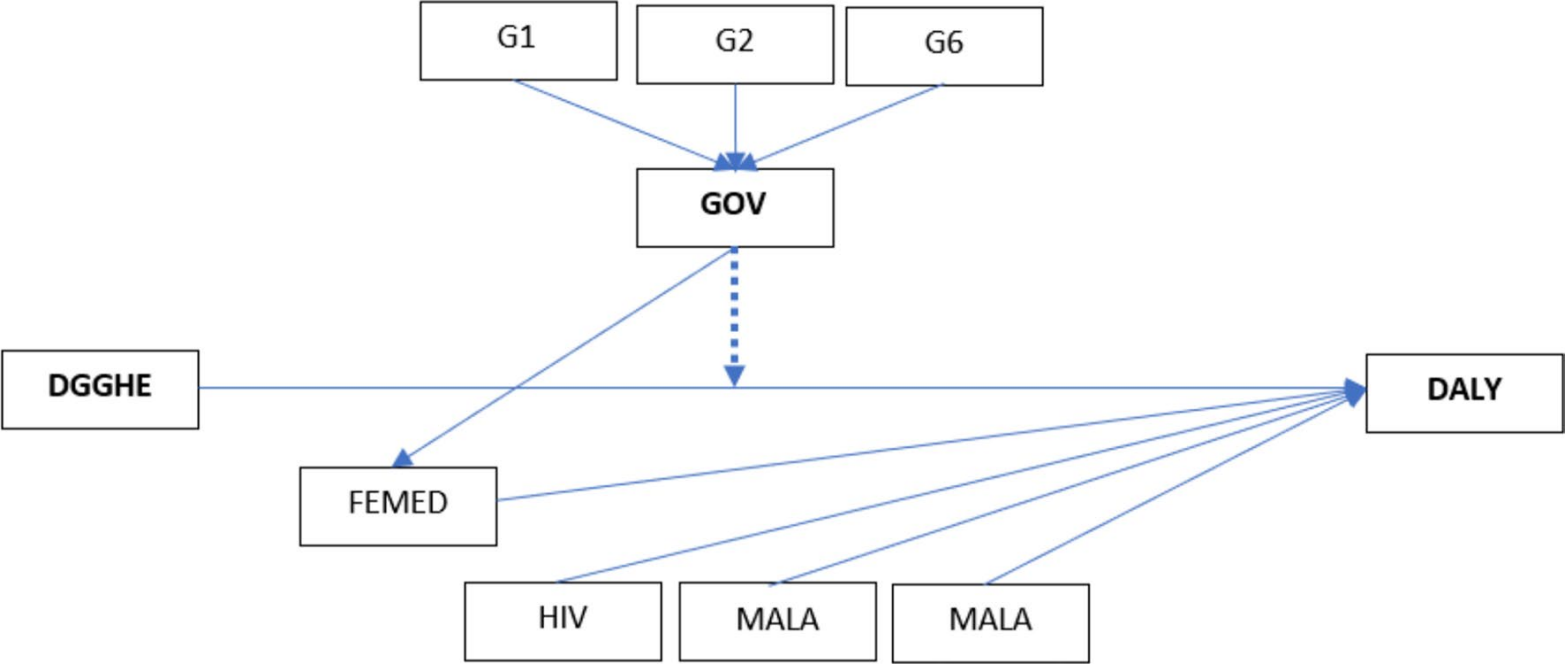
Model framework. DALY is the dependent construct; DGGHE is the independent construct; GOV is the moderator; and FEMED, HIV, MALA and TB are control constructs or variables. Control of corruption (G1), government effectiveness (G2) and voice accountability (G6) are indicators of GOV. The dotted line indicates the moderator effect on the relationship between DGGHE and DALY

The study model was built on the basis of Grossman’s model and extended to a macro-level health production function [17]. This model was created for data analysis using SmartPLS 3.3.8 software [63]. Figure 1 depicts the model framework used to test the study hypotheses. This model comprises five exogenous constructs (DGGHE, MALA, TB, HIV and GOV) and two endogenous constructs (DALY and FEMED). Governance is a second-order construct, with first-order construct indicators being control of corruption (G1), government effectiveness (G2) and voice accountability (G6) as selected indicators. All first-order constructs were measured employing multiple indicators. Paths from the exogenous to the endogenous variables provided a platform for analysis to determine support for hypotheses. According to the study hypotheses, a negative relationship was expected for the path from DGGHE to DALY and a negative one for the path from DGOV to DALY. All the components of government have been modelled as reflective constructs since it was anticipated that the included indicators would covary with one another, as they all pertain to the same subject; hence, they must have the same antecedents and outcomes [13].

### Data analysis

To achieve the study’s objective, two structural equation models were analysed: one excluding the moderator (ME model), which was used to test study hypothesis 1 and to prove the link on which the moderating effect is based [1], and another including the moderator (MI model) used to check research hypothesis 2. Following the study by Frank and Mcineka [19], the current study analysed the models by employing the PLS-SEM approach with the two procedures suggested by [25, 28], the assessment of the measurement model and the assessment of the structural model. In contrast to Frank and Mcineka’s study, this study went a step further by applying the two-stage approach to moderation.

The ME model is a reflective model that includes six lower-order constructs (LOC). In the first stage, as part of the measurement assessment, all the LOC were assessed for internal consistency, convergent validity and discriminant validity [25]. In the second stage, as part of the evaluation of the structural model and in accordance with the procedure suggested by [25, 28], the study model was examined for collinearity issues, significance and relevance of the path coefficients, degree of *R*^2^, effect size (*f*^2^), predictive relevance (*Q*^2^) and predictive power (*q*^2^).

The MI model is a reflective-reflective higher-order model with nine LOC, one higher-order construct (HOC) and an interaction term. The disjoint two-phase approach was applied to assess the HOC measurement model [64]. The HOC, that is, the GOV construct, was then assessed for internal consistency, convergent validity and discriminant validity.

On the basis of the MI model, the moderation analysis was conducted following the procedure suggested by Memon et al. (2019). This suggests focusing on the significance of the moderating effect on the basis of which the decision as to whether there is any moderating effect should be made, then calculating and reporting the effect size and, lastly, executing and reporting a simple slope plot for the visual inspection of the direction and strength of the moderating effect [47]. The assessment of this model’s structure followed the same procedure as the evaluation of the ME model.

All the study models were estimated using the bootstrapping procedure with 5000 bootstrap samples, using the No Sign Changes option, complete bootstrapping, bias corrected and accelerated bootstrap and two-tailed testing and as the standard settings for the PLS-SEM algorithm with the mean value replacement for missing values. The PLS-SEM algorithm was used for model estimation, while the bootstrapping procedure tested the significance of the PLS-SEM results. The significance of estimates was assessed considering a 5% significance level and the fact that the bias-corrected 95% confidence intervals for coefficient estimates should not include the null hypothesis value [25].

## Results

### Model assessment

#### Measurement model

**Fig. 2:**
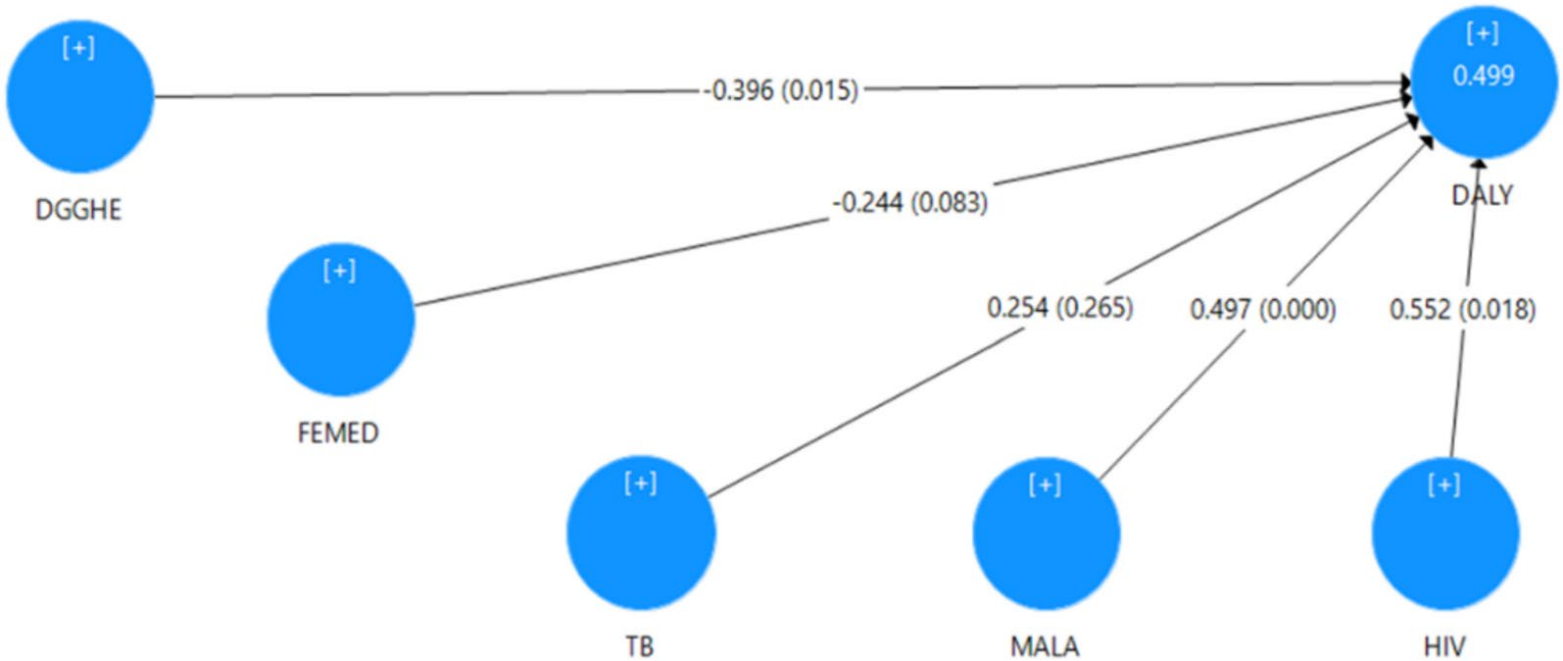
ME model estimates. Authors’ computation. Estimated path coefficients and corresponding *P*-values in brackets are presented on arrows. Estimated *R*^2^ is presented in the DALY circle

**Fig. 3:**
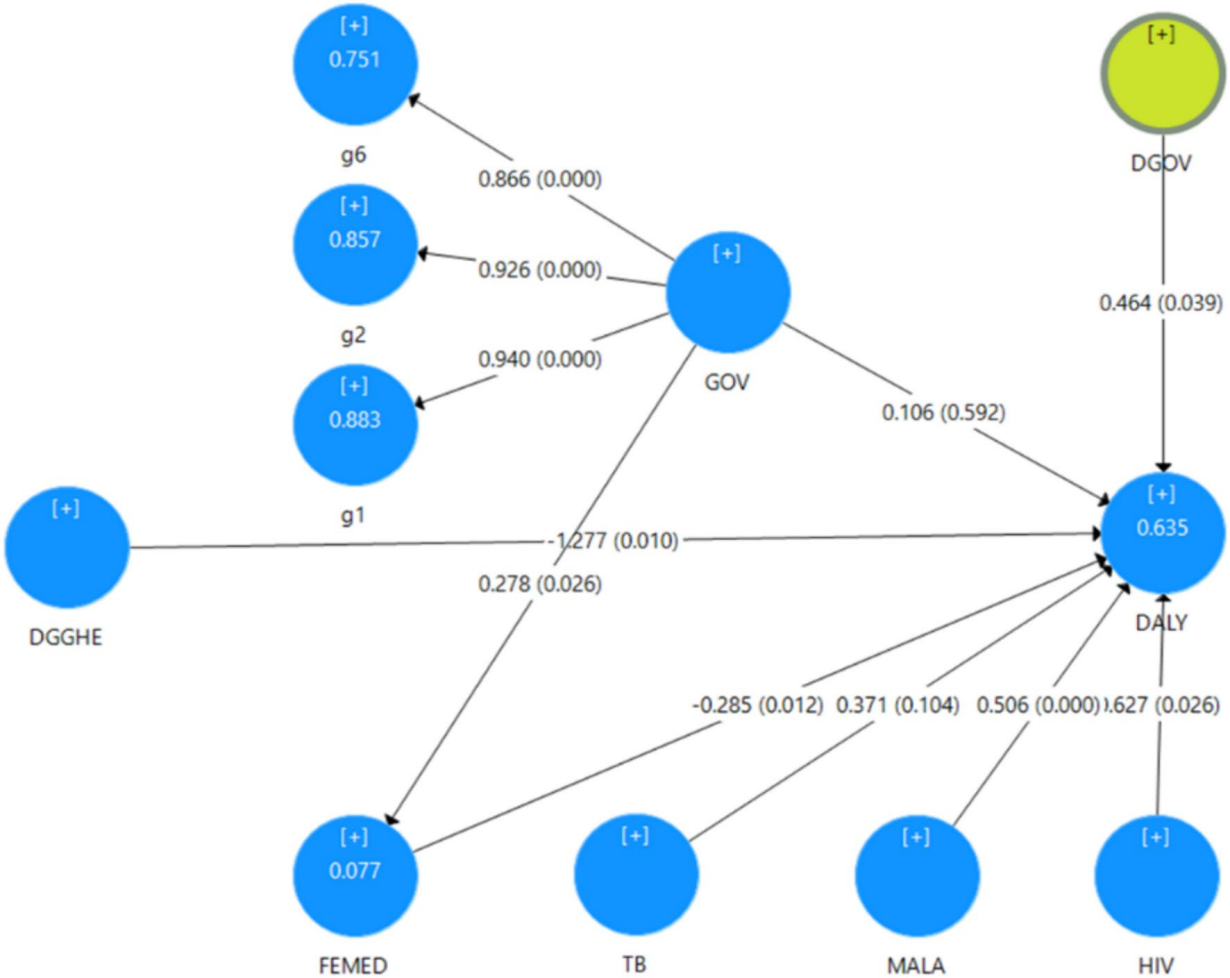
MI model estimates. Authors’ computation. Estimated path coefficients and corresponding *P*-values in brackets are presented on arrows. Estimated *R*^2^ are presented in the circles of the endogenous constructs DALY and FEMED. Coefficients of the paths towards GOV are factor loadings, while other coefficients represent path effects

The two study models, the ME model in Fig. 2 and the MI model in Fig. 3, were estimated and assessed. Findings from the measurement assessment are presented in Appendix 1a and 1b. Findings from the measurement assessment of the ME model presented in Appendix 1a inform composite reliability and Cronbach’s alpha of all the LOC greater than 0.70 and less than 1, indicating higher levels of reliability for the LOC. The outer loadings of the constructs and the average variance extracted (AVE) range from 0.876 to 1. This indicates a convergent validity for the LOC, as the common rule of thumb suggests for outer loadings and AVE values of 0.708 or higher [25]. The Heterotrait–Monotrait (HTMT)-based assessment reveals that, for all the LOC, the HTMT confidence intervals do not contain the value 1, indicating that the models exhibit discriminant validity. Findings from the measurement assessment of the MI model presented in Appendix 2a indicate the composite reliability and Cronbach’s alpha of 0.930 and 0.987, respectively, which also indicate higher levels of reliability for the HOC model. The outer loadings of the constructs (0.945, 0.960 and 0.799) and the average variance extracted (0.818) exceed 0.708, suggesting a convergent validity of the HOC. Finally, the HTMT confidence interval of the HOC does not contain the value 1, suggesting that the model presents discriminant validity. Following the measurement assessment of both MI models carried out above and ME, it can be concluded that the two models exhibit reliability, convergent validity and discriminant validity. Therefore, an assessment of their structural model was conducted.

#### Structural model

The structural model was assessed following the procedure from [25, 28]. The ME structural model’s assessment findings are presented in Tables 2, 3, 4, 5, 6, and 7. The collinearity among the predictor constructs is not a critical issue in the model. The results presented in Table 2 indicate that all variance inflation factor (VIF) values are clearly below the threshold of 5.

**Table 2:** Variance inflation factors

Constructs	VIF
DGGHE	2.026
FEMED	1.091
HIV	2.625
MALA	1.330
TB	2.109

Source: Authors’ computation

**Table 3:** Indicators of effect sizes

Paths	Path effect coefficients	T-statistics	*P*-values	95% confidence intervals	Significance (*P* < 0.05) ?
DGGHE → DALY	–0.396**	2.378	0.017	[−0.706, −0.059]	**Yes**
FEMED → DALY	−0.244	1.734	0.083	[−0.492, 0.078]	No
HIV → DALY	0.552**	2.379	0.057	[0.109, 1.012]	No
MALA → DALY	0.497***	3.631	0.000	[0.199, 0.745]	**Yes**
TB → DALY	0.254	1.126	0.260	[−0.203, 0.689]	No

Source: Authors’ computation. Note: The  results in bold indicate variables that are statistically significant, at a 5%  and 1 % levels,  in influencing DALY. **p < 0.05; ***p < 0.01

**Table 4:** *R*^2^ indicator

Construct	*R*^2^	T-statistic	*P*-value	95% confidence intervals
DALY	0.499***	4.889	**0.000**	**[0.222, 0.630]**

Source: Authors’ computation.  Note: The  results in bold indicate statistical significance at a 1% level for R-square. ***p < 0.05

**Table 5:** Indicators of effect sizes

Paths	Effect sizes (*f*^2^)	95% confidence intervals
DGGHE → DALY	0.155**	**[0.477, 0.281]**
FEMED → DALY	0.109**	**[0.201, 0.247]**
HIV → DALY	0.232	[−0.139, 0.319]
MALA → DALY	0.371	[−0.055, 0.451]
TB → DALY	0.061	[−0.710, 0.277]

Source: Authors’ computation. Note: The  results in bold indicate variables that are statistically significant,  at a 5% level,  in influencing DALY. **p < 0.05

**Table 6:** Indicators of predictive relevance

Constructs	SSO	SSE	Predictive relevance *Q*^2^ (= 1 − SSE/SSO)
DALY	301	156	0.482
DGGHE	301	301	
FEMED	301	301	
HIV	301	301	
MALA	301	301	
TB	301	301	

Source: Authors’ computation

**Table 7:** Predictive power indicators

Excluded construct	Predictive power (*q*^2^)
DGGHE	0.143
FEMED	0.098
HIV	0.224
MALA	0.349
TB	0.052

Source: Authors’ computation

Figure 2 presents estimates of the ME structural model, and Table 3 summarizes the findings from the model estimation taken from the report of the PLS-SEM algorithm and the bootstrapping procedure.

Assuming that the bias-corrected 95% confidence intervals do not include zero, only the paths coefficient effects DGGHE → DALY (−0.396; *P* = 0.017) and MALA → DALY (0.497; *P* = 0.000) are statistically significant. These results suggest that DGGHE and MALA only impact DALY in the SSA region. However, increased DGGHE decreases DALY, increased malaria incidence increases DALY, *ceteris paribus*. The *R*^2^ indicator for DALY (0.499) provided in Table 4 is significant as the corresponding bias-corrected 95% confidence interval does not include zero. This provides a moderate indication of explained variance, following the rule of thumb [28, 31].

As shown in Table 5, only the effect sizes of the paths DGGHE → DLY and FEMED → DALY are significant, as their bias-corrected 95% confidence intervals do not include zero. However, according to the guidelines for assessing *f*^2^, the effect size on DALY is small for FEMED and medium for DGGHE [12]. The *Q*^2^ values of DALY (0.482) shown in Table 6 are above zero, suggesting that the external constructs (DGGHE, FEMED, HIV, MALA and TB) have predictive relevance for the endogenous construct under examination (DALY).

The predictive powers *q*^2^ are computed using the following equation:

$${q}_{x\hspace{0.17em}\to \hspace{0.17em}y}^{2}=\frac{{Q}_{\text{included}}^{2}-{Q}_{\text{excluded}}^{2}}{1- {Q}_{\text{included}}^{2}}$$

where ${Q}_{\text{included}}^{2}$ results from the previous blindfolding estimation and ${Q}_{\text{excluded}}^{2}$ value is obtained from a model re-estimation after deleting a specific predecessor of that endogenous latent variable [25]. The predictive power indicators are presented in Table 7.

The results suggest that the exogenous constructs TB, FEMED and DGGHE have small predictive relevance for DALY, while HIV and MALA have medium predictive relevance for DALY.

The MI structural model’s assessment findings are presented in Tables 8, 9, 10, 11, and 12. In this model, indicators of VIF suggest that the collinearity among the predictor constructs is also not a critical issue, following the results in Table 8.

**Table 8:** Variance inflation factor for the MI model

Constructs	VIF
DGGHE	3.279
FEMED	1.210
GOV	2.059
HIV	2.635
MALA	1.330
TB	2.405

Source: Authors’ computation

**Table 9:** Indicators of effect sizes

Path	Path effect coefficients	T-statistics	*P*-values	95% confidence intervals
DGGHE → DALY	**−1.277**	2.588	0.010	[−2.308, −0.465]
DGOV → DALY	0.464**	2.064	0.039	[0.143, 1.060]
FEMED → DALY	−**0.285**	2.502	0.012	[−0.512, −0.056]
GOV → DALY	0.106	0.536	0.592	[−0.268, 0.518]
GOV → FEMED	0.278	2.227	0.026	[−0.023, 0.487]
HIV → DALY	0.627	2.228	0.026	[−0.001, 1.132]
MALA → DALY	0.506***	3.697	0.000	[0.185, 0.740]
TB → DALY	0.371	1.627	0.104	[−0.071, 0.768]

Source: Authors’ computation.  Note: The  results in bold indicate variables that are statistically significant,  at 5% and 1%  levels,  in influencing DALY. **p < 0.05; ***p < 0.01

**Table 10:** *R*^2^ indicator for MI model

Constructs	*R*^2^	T-statistics	*P*-values	95% confidence intervals
DALY	0.635	7.902	**0.000**	**[0.335, 0.730]**
FEMED	0.077	1.113	0.266	[0.001, 0.241]

Source: Authors’ computation

**Table 11:** Effect size indicator for MI model

Paths	Effect sizes (*f*^2^)	95% confidence intervals
DGGHE → DALY	0.509	[1.177, 1.706]
FEMED → DALY	0.180	[0.358, 0.340]
HIV → DALY	0.403	[−0.561, 0.700]
MALA → DALY	0.528	[0.260, 0.783]
TB → DALY	0.156	[−0.693, 0.426]
DGOV → DALY	0.368	[0.149, 0.749]
GOV → DALY	0.015	[−0.536, 0.321]
GOV → FEMED	0.084	[−0.520, 0.136]

Source: Authors’ computation

**Table 12:** Predictive relevance indicator for the MI model

Constructs	SSO	SSE	Predictive relevance *Q*^2^ (= 1 − SSE/SSO)
DALY	301	135	0.551
DGGHE	301	301	
FEMED	301	283	0.060
HIV	301	301	
MALA	301	301	
TB	301	301	
DGOV	43	43	
GOV	903	903	

Source: Authors’ computation

Figure 3 displays estimates of the MI structural Model, and Table 9 summarizes the results from the model estimation taken from the reports of the PLS-SEM algorithm and the bootstrapping procedure.

As can be seen in Table 9, assuming a 5% significance level and considering the 95% bias-corrected bootstrap confidence, all paths to DALY and FEMED are significant, except paths from GOV, HIV and TB to DALY. The interaction term (DGOV) is statistically significant (0.464; *P* = 0.039) and has a positive effect on DALY, whereas the simple effect of DGGHE on DALY (−1.277; *P* = 0.010) is negative.

Results from Table 10 suggest significant *R*^2^ indicators of 0.635 and 0.077 for DALY and FEMED, respectively. While the *R*^2^ for FEMED is insignificant, that of DALY is significant, suggesting a moderate indication of explained variance.

Estimating the ME and MI models shows that the inclusion of the moderator and interaction term in the MI model slightly increases the *R*^2^ of the study model from 0.499 to 0.635. Therefore, it is beneficial to include the moderator and interaction term in the model from a prediction standpoint.

The effect sizes *f*^2^ of all paths on DALY are significant, as shown in Table 11, although differing in magnitude. The *Q*^2^ values of 0.482 and 0.060 for DALY and FEMED, respectively, shown in Table 12, are above zero, suggesting that the external constructs (DGGHE, FEMED, HIV, MALA, GOV, DGOV and TB) have predictive relevance for DALY, and GOV has predictive relevance for FEMED. The moderation assessment subsection carries out the model significance and relevance assessment.

The concept of model fit, as understood from the CB-SEM approach, is not fully transferable to the PLS-SEM approach since the method estimates model parameters with a different objective. Despite this, researchers have produced several PLS-SEM-based model fit measures, including SRMR, in the early stages of development [25]. The SRMR indexes of MI and ME models are presented in Appendix 1b. The values of 0.04 and 0.07 lay in the acceptable range for the SRMR index of 0–0.08 (Hu and Bentler 1999), suggesting the models are parsimonious.

### Moderation assessment

Although the significance of DGGHE is of interest as it establishes the relationship that is moderated, the primary interest is in the significance of the interaction term (DGOV) because it allows us to conclude that the moderator has a significant moderating effect on the relationship between DGGHE and DALY. Findings from Table 9 indicate coefficients of −1.277 (*P* = 0.010) and 0.464 (*P* = 0.039) for DGGHE and DGOV, respectively. The significance of the two parameters is confirmed by the 95% bias-corrected bootstrap confidence intervals of DGGHE and DGOV of [−2.308, −0.465] and [0.143, 1.060], respectively. Jointly, these results indicate the negative impact of DGGHE on DALY of −1.277 for the average level of governance. For higher levels of governance (for example, GOV is increased by one standard deviation unit), this negative impact decreases to −0.813 (−1.277 + 0.464).

**Fig. 4:**
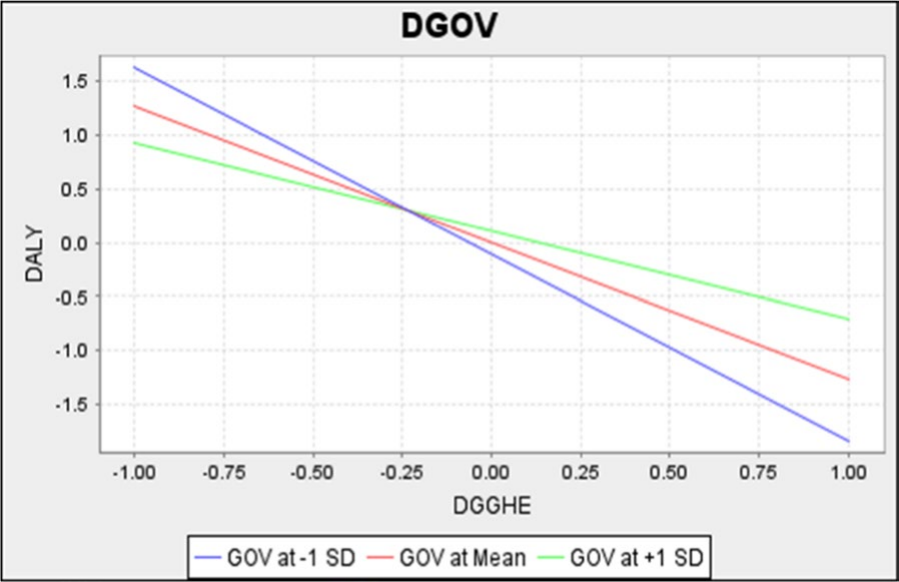
Simple slope analysis. Source: Authors’ computation

On the contrary, for lower levels of GOV (for example, GOV is decreased by one standard deviation point), the impact becomes −1.741 (−1.277–0.464). These findings indicate that the negative relationship between DGGHE and DALY is dampened by high levels of governance and strengthened by low levels of governance, therefore suggesting that governance exerts a weakening substituting interaction effect on the relationship between DGGHE and DALY [20]. The simple slope plot (Fig. 4) illustrates the impact of the two-way interaction to better understand the moderator analysis’s outcomes. The three lines in Fig. 4 represent the relationship between DGGHE (*x*-axis) and DALY (*y*-axis). The red, blue and green lines represent this relationship for the average, lower and higher levels of governance.

The relationship between DGGHE and DALY is negative for all three lines, as indicated by their negative slope. Nevertheless, the line representing a lower level of governance is steeper than that representing a higher level. This suggests that higher levels of governance are related to lower levels of DALY. For the completeness of the presentation of the results, the study addresses the moderator’s effect size *f*^2^. Results in Table 11 suggest that the interaction term’s *f*^2^ effect size is 0.368. This value indicates a large effect [25].

## Discussion

Poor population health is detrimental in SSA, where health financing is ineffective and poor governance is a serious issue. Therefore, the need arose for this paper to assess the effectiveness of PHS in SSA and, more importantly, the role of governance in the effectiveness of PHS. Two hypotheses were tested: first, to see whether there is a meaningful link between public spending and health, and second, to determine whether this affects governance. The results of structural equation modelling provided evidence that DGGHE significantly reduced DALY in SSA countries and that the country governance (control of corruption, government effectiveness and voice accountability) moderated this effect. The empirical analysis revealed that, on average, the association between DALY and DGGHE is statistically significant, with a negative impact of DGGHE on DALY across the 43 SSA countries for the study period. Specifically, an increase of 1 standard deviation in DGGHE is associated with a decrease of 1.277 standard deviations in DALY. These results support the study hypothesis 1, which suggests an increase in DGGHE is associated with a reduction of DALY. This result was expected because most SSA healthcare services are publicly owned and funded.

Moreover, compared with privately held facilities, publicly owned facilities’ pricing is accessible for low-income populations. In this context, DGGHE is more likely to affect a larger percentage of the population than private health spending. The results in this study contrast Nicholas et al. (2016) and line up with Novignon and Lawanson [51], Gupta et al. [23] and Farag et al. [16] but solidify the evidence of one of the findings in this literature by using DALY, which is a more comprehensive measure of health output than mortality used mainly by previous studies.

The current empirical analysis also reveals that DGGHE moderates the role of public financing in producing health output. The moderating effect coefficient is statistically significant and consistent with hypothesis 2: governance moderates the relationship between DGGHE and DALY. The results suggest that the negative impact of DGGHE on DALY is moderated by the country’s governance such that the relationship (slope) becomes weaker as governance improves. In other words, the negative relationship between DGGHE and DALY is dampened by high levels of governance and strengthened by low levels of governance. Therefore, governance exerts a weakening substituting interaction effect [20] on the relationship between DGGHE and DALY, as the conditional DGGHE–DALY relationship dissipates as governance increases. The study results depict disordinal interactions (Fig. 4). The moderating effects differ on either side of the crossing point of lines. Before the cross point, the moderating effect is higher for lower levels of governance and lower for higher levels of governance. After the cross point, the opposite is true. This situation indicates that countries in the first stage of governance implementation record substantial reductions in DALY due to increases in DGGHE compared with those with good governance seeking improvement. For example, the change in the levels of corruption is substantial following the first stage of implementing governance because of the people’s fear of the implemented measures. Over time, individuals develop circumvention strategies that undermine the effectiveness of anti-corruption measures.

The study pointed out other factors, such as female education, the prevalence of HIV/AIDS, the incidence of malaria and the incidence of tuberculosis, suggesting that these factors cannot be overlooked as contributing to the level of DALY. Several studies have extensively documented the benefits of enhancing female education, emphasizing its importance to the population’s health [49]. Moreover, HIV/AIDS, tuberculosis and malaria are among the leading causes of death and major risk factors contributing to the disease burden in SSA [53, 66].

The influence of governance on the effectiveness of public spending on health output in SSA has been limited and needs more research. While extensive research has been conducted on health spending and outcomes, few studies have focused on the association between DGGHE and DALY. DALY has more information than just mortality, as it accounts for disability even for surviving members and captures other aspects of health besides longevity. In addition, this study also contributes to the literature by considering governance as a stimulus that may influence the effectiveness of PHS. Good governance is crucial for a country’s social and economic development, stability, security and inclusive progress [46]. Lastly, this study contributed by using an empirical PLS-SEM that has not been used extensively on SSA data. The use of PLS-SEM in this study follows recommendations of various researchers that the PLS approach should be applied more frequently because it can model latent constructs under conditions of non-normality in small to medium sample sizes [25], maximizing the explained variance of the endogenous variables and predicting values for latent constructs using multiple regressions [11].

Finally, it must be noted that although the current study’s findings support increasing DGGHE and improving the country’s governance, these improvements may only be necessary but insufficient. Achieving a decline in DALY may depend on some other factors not included in this study because of the lack of data at the country level. In addition, the study used a negative indicator of health outcome (DALY) as a proxy for PHO. Using a positive indicator of health outcomes, such as healthy life expectancy, may have provided substantial insights. Moreover, the study model did not capture the dynamic effects that may have brought other insights. For example, the weaker effects of DGGHE among countries with higher levels of governance may mean the effects of increasing governance have reached their end-point of impact. This situation could be well depicted by investigating the dynamic aspect of the topic. Although these limitations may serve as the basis for future research, they do not undermine the current study’s findings.

## Conclusion

In the SSA region, one of the challenges governments encounter is translating government spending on health into PHO to improve the population’s health. This study sought to test two hypotheses. The evidence suggested that DGGHE significantly reduces DALY across all the models specified in the study and that governance improves the effect of DGGHE on DALY with bigger improvement among countries with better governance. These findings provide evidence that good governance is crucial to the effectiveness of PHS in SSA nations. Therefore, sub-Saharan African countries should improve governance quality in planning health-spending-based interventions to improve population health.

## Data Availability

The data supporting this study’s findings are included as supplementary materials in this submission.

## Appendix 1a: Results of measurement assessment of ME model

**Table:**

Constructs	Indicators	Outer loadings	Cronbach’s alpha	Composite reliability	Average variance extracted	Paths	HTMT ratio	0.05	0.95
DALY	daly22013	0.995	0.999	0.999	0.995	DGGHE → DALY	0.128	0.029	0.312
	daly22014	0.995				FEMED → DALY	0.207	0.057	0.409
	daly22015	0.999				FEMED → DGGHE	0.032	0.016	0.029
	daly22016	0.999				HIV → DALY	0.229	0.028	0.478
	daly22017	0.999				HIV → DGGHE	0.650	0.437	0.806
	daly22018	0.998				HIV → FEMED	0.177	0.047	0.339
	daly22019	0.996				MALA → DALY	0.408	0.146	0.662
DGGHE	dgghe17_2013	0.997	0.999	0.999	0.992	MALA → DGGHE	0.476	0.323	0.595
	dgghe17_2014	0.999				MALA → FEMED	0.120	0.057	0.154
	dgghe17_2015	0.995				MALA → HIV	0.407	0.150	0.596
	dgghe17_2016	0.994				TB → DALY	0.265	0.033	0.503
	dgghe17_2017	0.997				TB → DGGHE	0.574	0.273	0.780
	dgghe17_2018	0.994				TB → FEMED	0.038	0.022	0.033
	dgghe17_2019	0.994				TB → HIV	0.707	0.509	0.822
FEMED	femed2013	0.938	0.976	0.980	0.876	TB → MALA	0.313	0.094	0.545
	femed2014	0.969				g1 → DALY	0.219	0.042	0.446
	femed2015	0.967				g1 → DGGHE	0.539	0.310	0.706
	femed2016	0.952				g1 → FEMED	0.262	0.081	0.487
	femed2017	0.926				g1 → HIV	0.312	0.056	0.560
	femed2018	0.901				g1 → MALA	0.314	0.121	0.547
	femed2019	0.897				g1 → TB	0.078	0.050	0.112
G1	g1cor2013	0.976	0.996	0.996	0.974	g2 → DALY	0.297	0.092	0.504
	g1cor2014	0.986				g2 → DGGHE	0.638	0.433	0.777
	g1cor2015	0.994				g2 → FEMED	0.238	0.084	0.436
	g1cor2016	0.993				g2 → HIV	0.394	0.144	0.625
	g1cor2017	0.993				g2 → MALA	0.274	0.086	0.507
	g1cor2018	0.988				g2 → TB	0.153	0.074	0.380
	g1cor2019	0.977				g2 → g1	0.847	0.747	0.908
G2	g2eff2013	0.977	0.996	0.996	0.974	g6 → DALY	0.100	0.026	0.282
	g2eff2014	0.985				g6 → DGGHE	0.407	0.118	0.573
	g2eff2015	0.989				g6 → FEMED	0.238	0.078	0.457
	g2eff2016	0.993				g6 → HIV	0.154	0.026	0.382
	g2eff2017	0.994				g6 → MALA	0.184	0.041	0.416
	g2eff2018	0.992				g6 → TB	0.087	0.025	0.221
	g2eff2019	0.979				g6 → g1	0.717	0.489	0.866
G6	g6voa2013	0.973	0.994	0.995	0.967	g6 → g2	0.678	0.462	0.812
	g6voa2014	0.986							
	g6voa2015	0.988							
	g6voa2016	0.992							
	g6voa2017	0.993							
	g6voa2018	0.981							
	g6voa2019	0.971							
HIV	hivprev2013	0.999	1.000	1.000	0.999				
	hivprev2014	1.000							
	hivprev2015	1.000							
	hivprev2016	1.000							
	hivprev2017	1.000							
	hivprev2018	1.000							
	hivprev2019	0.999							
MALA	malinc2013	0.929	0.988	0.990	0.931				
	malinc2014	0.973							
	malinc2015	0.989							
	malinc2016	0.995							
	malinc2017	0.925							
	malinc2018	0.970							
	malinc2019	0.972							
TB	tb2013	0.965	0.995	0.996	0.972				
	tb2014	0.981							
	tb2015	0.994							
	tb2016	0.999							
	tb2017	0.997							
	tb2018	0.990							
	tb2019	0.975							

## Appendix 2a: Results of measurement assessment of MI model

**Table:**

Constructs	Indicators	OUTER LOADINGS	Cronbach’s alpha	Composite reliability	Average variance extracted	Paths	HTMT ratio	0.05	0.95
DALY	daly22013	0.995	0.999	0.999	0.995	DGGHE → DALY	0.128	0.030	0.261
	daly22014	0.995				FEMED → DALY	0.207	0.050	0.423
	daly22015	0.999				FEMED → DGGHE	0.032	0.017	0.030
	daly22016	0.999				GOV → DALY	0.239	0.083	0.460
	daly22017	0.999				GOV → DGGHE	0.610	0.342	0.751
	daly22018	0.998				GOV → FEMED	0.285	0.108	0.491
	daly22019	0.996				HIV → DALY	0.229	0.038	0.472
DGGHE	dgghe17_2013	0.997	0.999	0.999	0.992	HIV → DGGHE	0.650	0.420	0.793
	dgghe17_2014	0.999				HIV → FEMED	0.177	0.050	0.340
	dgghe17_2015	0.995				HIV → GOV	0.330	0.119	0.564
	dgghe17_2016	0.994				MALA → DALY	0.408	0.111	0.625
	dgghe17_2017	0.997				MALA → DGGHE	0.476	0.301	0.589
	dgghe17_2018	0.994				MALA → FEMED	0.120	0.050	0.165
	dgghe17_2019	0.994				MALA → GOV	0.297	0.113	0.501
FEMED	femed2013	0.947	0.976	0.980	0.877	MALA → HIV	0.407	0.139	0.594
	femed2014	0.971				TB → DALY	0.265	0.042	0.536
	femed2015	0.965				TB → DGGHE	0.574	0.263	0.771
	femed2016	0.957				TB → FEMED	0.038	0.025	0.035
	femed2017	0.914				TB → GOV	0.118	0.048	0.203
	femed2018	0.891				TB → HIV	0.707	0.535	0.820
	femed2019	0.906				TB → MALA	0.313	0.090	0.527
GOV	g1	0.945	0.897	0.930	0.818				
	g2	0.960							
	g6	0.799							
HIV	hivprev2013	0.999	1.000	1.000	0.999		**ME model**	**MI model**	
	hivprev2014	1.000							
	hivprev2015	1.000				SRMR	0.04	0.07	
	hivprev2016	1.000							
	hivprev2017	1.000							
	hivprev2018	1.000							
	hivprev2019	0.999							
MALA	malinc2013	0.929	0.988	0.990	0.931				
	malinc2014	0.973							
	malinc2015	0.989							
	malinc2016	0.995							
	malinc2017	0.925							
	malinc2018	0.970							
	malinc2019	0.972							
TB	tb2013	0.965	0.995	0.996	0.972				
	tb2014	0.981							
	tb2015	0.994							
	tb2016	0.999							
	tb2017	0.997							
	tb2018	0.990							
	tb2019	0.975							

## Abbreviations

PHS: Public health spending
PHO: Population health outcomes
DGGHE: Domestic general government health expenditure
DALY: Disability-adjusted life years
PLS-SEM: Partial least squares structural equation modelling
SSA: Sub-Saharan Africa
THE: Total health expenditure
PHE: Public health expenditures
ME model: Estimated model
MI model: Interaction models (moderation model)
